# Nomenclature and typification of *Polygala nicaeensis* (Polygalaceae) and its infraspecific taxa

**DOI:** 10.3897/phytokeys.270.174954

**Published:** 2026-02-02

**Authors:** Francesca Manconi, Lorenzo Peruzzi

**Affiliations:** 1 PLANTSEED Lab, Department of Biology, University of Pisa, Pisa, 56126, Italy University of Pisa Pisa Italy

**Keywords:** Lectotype, Mediterranean flora, neotype, protologue, taxonomy

## Abstract

*Polygala nicaeensis* is a circum-Mediterranean species with uncertain infraspecific taxonomy, currently split into eight subspecies. As a first step of an ongoing taxonomic and systematic study, we have identified and studied 29 nomenclatural types (of which 23 lectotypes and one second-step neotype are designated here), to fix the application of all the names connected to this species. We also re-defined the year, place and authorship of first publication of *P. nicaeensis* itself, whose correct name is *P. nicaeensis* Risso ex Bray. Based on the types studied here, the currently widely-accepted *P. nicaeensis* subsp. mediterranea Chodat, widespread in the Mediterranean, becomes a heterotypic synonym of *P. nicaeensis* s.str. Finally, *P. nicaeensis* subsp. corsica and *P. pedemontana* E.P.Perrier & B.Verl. are confirmed as heterotypic synonyms, that apply to a species occurring in Corsica and Western Alps, whose correct priority name is *P. corsica* Boreau.

## Introduction

*Polygala nicaeensis* is a circum-Mediterranean species with uncertain infraspecific taxonomy (Tison et al. 2014; Pignatti 2018). This species belongs to the cosmopolitan genus *Polygala*, one of “big genera” of angiosperms deserving in-depth studies (Moonlight et al. 2024). Indeed, this genus currently includes between 562 and 673 accepted species/subspecies (Pastore et al. 2019; WFO 2025).

Eight subspecies are currently accepted within *Polygala nicaeensis* (Arrigoni 2014; Tison and de Foucault 2014; Euro+Med 2025): *P. nicaeensis* subsp. caesalpini (Bubani) McNeill, distributed in Spain; *P. nicaeensis* subsp. corsica (Boreau) P.Graebn., distributed in Corsica and Peninsular Italy; *P. nicaeensis* subsp. gariodiana (Jord. & Fourr.) Chodat, endemic to France (and doubtfully reported from Italy); *P. nicaeensis* subsp. italiana (Chodat) Arrigoni, endemic to the northern Italian Peninsula between Tuscany, the Republic of San Marino and Umbria; *P. nicaeensis* subsp. mediterranea Chodat, showing a wide circum-Mediterranean distribution; *P. nicaeensis* subsp. nicaeensis, with a scattered Mediterranean distribution; *P. nicaeensis* subsp. peninsularis Arrigoni, endemic to Peninsular Italy; *P. nicaeensis* subsp. tomentella (Boiss.) Chodat, endemic to Greece. These subspecies show large areas of morphological and geographical overlap. Sometimes, variation is observed within the same individual depending on its phenology and developmental stage (Pignatti 2019). Many of the names connected to *P. nicaeensis* and its subspecies are not yet typified. Accordingly, as a first step of an ongoing taxonomic and systematic study on this species, we have identified and studied here 29 nomenclatural types, including 23 formal type designations.

## Materials and methods

For the typification of *Polygala nicaeensis* and connected names, we searched this name and putative synonyms in Arrigoni (2014), Euro+Med (2025), IPNI (2025), POWO (2025) and WFO (2025). After this preliminary search, given some inconsistencies about the place of first valid description of *P. nicaeensis* amongst databases, we checked for the name *P. nicaeensis* in all the publications of the first half of 19^th^ century. By studying the protologues, we searched the original material in several European Herbaria (ANG, BC, BE, BESA, BR, BRNM, CHE, CLF, COI, FI, G, GAP, GE, LY, LE, M, MPU, P, PI, REG, SOM, WU, acronyms follow Thiers (2025)). Then, we proceeded with typifications according to the rules of the International Code of Nomenclature for Algae, Fungi and Plants (Turland et al. (2025); ICN hereafter). The names are listed in alphabetical order. Illustrations of the designated types are shown only for those specimens not available online in digitised collections.

### Typification of the names

#### Polygala caesalpini

Taxon classificationPlantaeFabalesPolygalaceae

Bubani, Fl. Pyren. 3: 283. 1901

DB1EAD83-C14D-59DF-A84D-76E36C9A4564

Fig. 1

≡ Polygala nicaeensis subsp. caesalpini (Bubani) McNeill, Feddes Repert. 79: 32. 1968.

##### Type.

Spain. “in Pyren. Merid Catalan Humid. Ad Gerona”, 15 May 1851, *P. Bubani* s.n. (lectotype, designated here: UNIGE-HGE 036582!).

**Figure 1.**
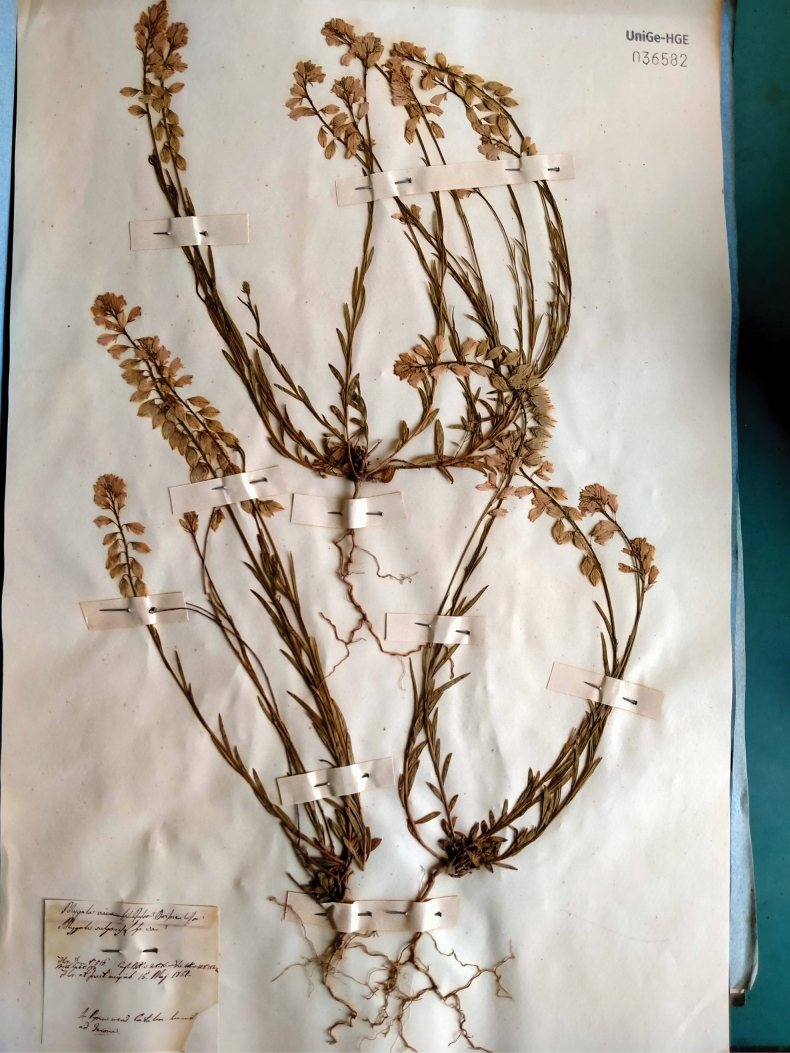
Lectotype of *Polygala caesalpini* Bubani conserved in GE. Reproduced with the permission of the Herbarium of the University of Genova.

In the protologue, Bubani (1901) mentions three syntypes: one specimen at page 175 of the Herbarium Cesalpino and two specimens collected by Bubani himself. The Herbarium of Andrea Cesalpino is preserved at FI! This specimen is very old and some parts are missing. According to Stafleu and Cowan (1976), the collections by Bubani are conserved at GE. In this Herbarium, we traced the two specimens mentioned in the protologue (UNIGE-HGE 036582!, UNIGE-HGE 036583!), one collected in Gerona and the other “supra Salsona”, each bearing three individuals. The specimen from Girona, more complete and better conserved, is selected here as lectotype. All the three traced specimens match the protologue and correspond to the current application of the name as *P. nicaeensis* subsp. caesalpini (Heywood 1968; McNeill 1968). Paiva (2007) accepts the name *P. nicaeensis* subsp. gerundensis (O.Bolòs & Vigo) Á.M.Hern. for this subspecies; however, it is a later heterotypic synonym (see below).

#### Polygala corsica

Taxon classificationPlantaeFabalesPolygalaceae

Boreau, Mém. Soc. Acad. Maine Loire 1: 87. 1857

1587B0C9-0AE1-5CD9-BE80-D908596B62A5

≡ Polygala nicaeensis var. corsica (Boreau) Chodat, Bull. Trav. Soc. Bot. Genève 5: 179. 1889 ≡ Polygala vulgaris “proles” corsica (Boreau) Rouy & Foucaud, Fl. France 3: 72. 1896 ≡ Polygala vulgaris f. corsica (Boreau) Paol. in Fiori & al., Fl. Anal. Italia 2: 230. 1901 ≡ Polygala nicaeensis subsp. corsica (Boreau) Graebn. in Asch. & Graebn., Syn. Mitteleur. Fl. 7: 337. 1916.

##### Type.

France. “Corse: Rogliano, rochers”, 4 July 1854, *Revelière* 42 (Lectotype, designated here: ANG037772!).

In the protologue, Boreau (1857) mentions “les plantes recuillies en Corse par M. E. Revellière”. Based on the indications found in Stafleu and Cowan (1976), we found a pertinent specimen in ANG. This specimen, collected by Revelière in 1854, is part of the Herbarium Boreau. This specimen is clearly original material, suitable for lectotypification. This is particularly relevant, since Chodat (1889: 451) indicated a specimen collected by Mabille in 1867 and conserved in P as “type”. However, this must be interpreted as a neotype designation, that is superseded by our lectotype designation (Art. 9.13 of the ICN). The lectotype matches the protologue. Based on the morphological features of the lectotype, *P. corsica* is very similar to the lectotype of *P. pedemontana* (see below). The only observed difference concerns the smaller width of the wings, i.e. the two larger lateral, petaloid, sepals (≤ 4.5 mm in the former, > 4.5 mm in the latter), that, however, show the same pattern of vein with anastomoses. In other words, the size of wings in *P. corsica* is similar to that of *P. comosa* Schkuhr, but this latter species shows nerves without anastomoses (Tison and de Foucault 2014). Accordingly, we consider *P. corsica* and *P. pedemontana* as heterotypic synonyms. This confirms what was previously suggested by other authors, that either synonymised (Jeanmonod and Gamisans 2013) or included (Tison and de Foucault 2014), this taxon should be placed under *P. pedemontana*. However, it should also be highlighted that the priority name at species level for this unit of diversity is *P. corsica* and not the later *P. pedemontana*.

#### Polygala coursiereana

Taxon classificationPlantaeFabalesPolygalaceae

Pomel, Bull. Soc. Sci. Phys. Algérie 11(7): 211 1874

00DE292B-8550-5528-AC96-9ED9B7C6E376

Fig. 2

≡ Polygala nicaeensis var. coursiereana (Pomel) Batt. in Batt. & Trab., Fl. Algérie, 1: 106. 1888.

##### Type.

ALGERIA. “Fort de l’eau”, s.d., *Coursière* s.n. (lectotype, designated here: MPU005110!).

**Figure 2.**
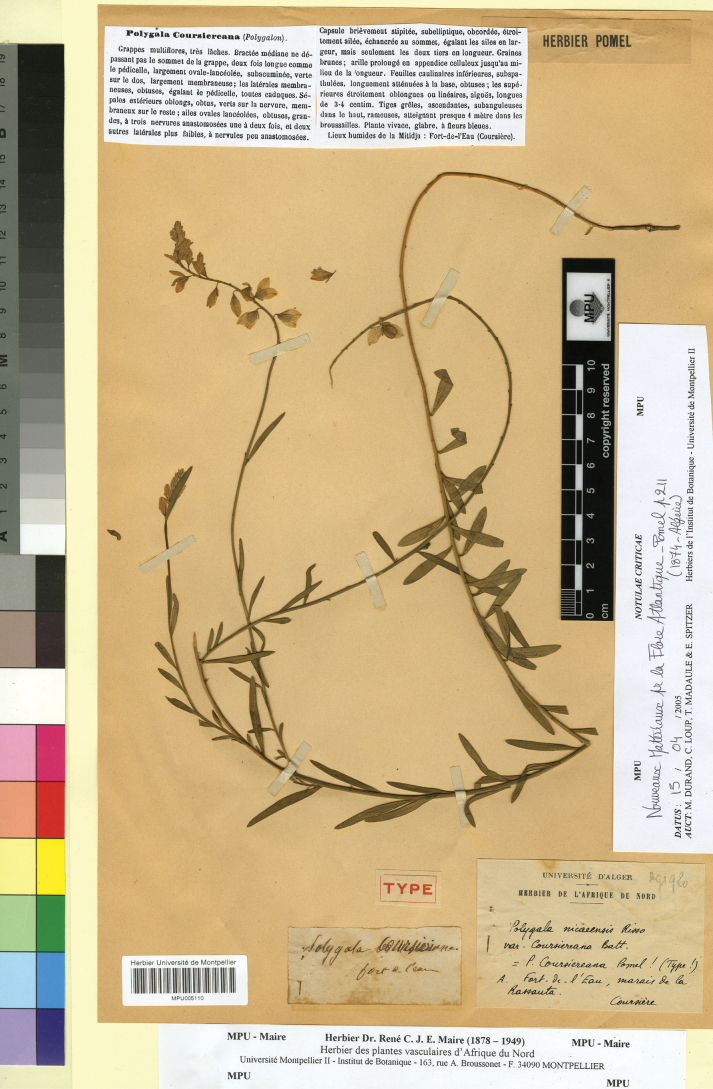
Lectotype of *Polygala coursiereana* Pomel in MPU. Reproduced with the permission of the Université de Montpellier – MPU Herbarium.

In the prologue, Pomel (1874) only mentions “*Fort de l’eau*” as place of collection. After searching for specimens in the various herbaria cited in Stafleu and Cowan (1983), we found a specimen in the Herbarium Pomel at MPU and collected by Coursière, which is original material, suitable for lectotypification. The specimen is in good conservation condition and a fruit, the wings and some corollas are clearly visible. The lectotype matches the protologue. Based on the morphological features of the lectotype, *P. coursiereana* is a heterotypic synonym of *P. nicaeensis* s.str., as circumscribed in this study, i.e. a taxon combining the morphological features and distribution of the former *P. nicaeensis* subsp. nicaeensis and *P. nicaeensis* subsp. mediterranea Chodat (see below).

#### Polygala gariodiana

Taxon classificationPlantaeFabalesPolygalaceae

Jord. & Fourr. in Verlot, Cat. Pl. Vasc. Dauphiné: 392. 1872

A6A2E18E-224B-529A-8E10-08296D70A9B9

≡ Polygala comosa var. gariodiana (Jord. & Fourr.) A.W.Benn, J. Bot. 16: 272. 1878. ≡ Polygala nicaeensis subsp. gariodiana (Jord. & Fourr.) Chodat, Bull. Trav. Soc. Bot. Genève 5: 180. 1889 ≡ Polygala vulgaris “proles” gariodiana (Jord. & Fourr.) Rouy & Foucaud, Fl. France 3: 72. 1896 ≡ Polygala vulgaris f. gariodiana (Jord. & Fourr.) Paol. in Fiori & al., Fl. Anal. Italia 2: 230. 1901.

##### Type.

France. “Lazer (entre Ventavon et Lavagne)”, 1 June 1869, *s. coll*. s.n. (lectotype, designated here: GAP065518!).

In the protologue (Verlot 1872), a gathering by H. Gariod is mentioned. We found a couple of specimens collected by Gariod, in GAP and in BR. The latter specimen (BR000000526922!) is less complete, so that we selected the specimen GAP065518! as the lectotype. This specimen is complete and in good conservation condition and both fruits and corolla tubes are visible. All the original material match the protologue. Both specimens correspond to the description given in the protologue and confirm the current application of the name as *P. nicaeensis* subsp. gariodiana (McNeill 1968; Arrigoni 2014).

#### Polygala mediterranea var. jordanovii

Taxon classificationPlantaeFabalesPolygalaceae

Kožuharov & A.V.Petrova, Fl. Nar. Republ. Bulgariya 7: 493. 1979

5480A8A3-9F29-5185-A7C4-D220D80F0714

##### Type.

Bulgaria. “Mt. Slavjanka, Pariliskj dol, in declivibus herbosis orientalis ad 1200 m s.m.”, 17 July 1957, *V. Velcev, S. Petrov, S. Gancev* s.n. (holotype: SOM104634!).

Based on the morphological features of the holotype, *P. mediterranea* subsp. jordanovii is a heterotypic synonym of *P. nicaeensis* s.str. as circumscribed in this study, i.e. a taxon combining the morphological features and distribution of the former *P. nicaeensis* subsp. nicaeensis and *P. nicaeensis* subsp. mediterranea Chodat (see below).

#### Polygala mediterranea var. parilica

Taxon classificationPlantaeFabalesPolygalaceae

Kožuharov & Petrova, Fl. Nar. Republ. Bulgariya 7: 492. 1979

2552A038-6B9E-5233-B282-FC8DD4B9EA4D

##### Type.

Bulgaria. “Mt. Rhodopy occidentalis, in declivibus australibus calcareis intra l. d. “Belgica” et Longurlii ad riv Suisuza, 1600 m s.m.”, 10 July 1930, *D. Jordanov* s.n. (holotype: SOM50240!).

Based on the morphological features of the holotype, *P. mediterranea* subsp. jordanovii is a heterotypic synonym of *P. nicaeensis* s.str. as circumscribed in this study, i.e. a taxon combining the morphological features and distribution of the former *P. nicaeensis* subsp. nicaeensis and *P. nicaeensis* subsp. mediterranea Chodat (see below).

#### Polygala nemorivaga

Taxon classificationPlantaeFabalesPolygalaceae

Pomel, Bull. Soc. Sci. Phys. Algérie 12: 337. 1875

CF3D3DE5-63C3-588A-BA1B-308D6786003D

≡ Polygala nicaeensis var. nemorivaga (Pomel) Bonnet in Bonnet & Barr., Expl. Sci. Tunisie, Cat. Pl.: 46. 1896.

##### Type.

Algeria. “du Filfilla a Bon Maiba”, 24 May 1874, *A.N. Pomel* s.n. (lectotype, designated here: MPU005613!).

In the protologue, Pomel (1874) mentions “*Clarières herbeuses des bois: massifs de Collo, du Filfila*” as source of the material studied. We traced three specimens, one bearing four individuals (MPU005613), one bearing three individuals (MPU005616) and one (P00375236) bearing three individuals, that are all original material. We selected the sample MPU005613 as lectotype, because the information reported on the label is the most complete, with collector and collection date visible. The lectotype matches the protologue. Based on the morphological features of the lectotype, *P. nemorivaga* is a heterotypic synonym of *P. nicaeensis* s.str. as circumscribed in this study, i.e. a taxon combining the morphological features and distribution of the former *P. nicaeensis* subsp. nicaeensis and *P. nicaeensis* subsp. mediterranea Chodat (see below).

#### Polygala nicaeensis

Taxon classificationPlantaeFabalesPolygalaceae

Risso ex Bray, Flora 1(supplement): 14. 1818

8F0BF311-5D2A-58B1-BBA3-9E7CF8423DBE

≡ Polygala nicaeensis Risso ex W.D.J.Koch, Syn. Fl. Germ. Helv.: 92. 1836, isonym (Art. 6.3 Note 2 of the ICN) ≡ Polygala nicaeensis (Risso) ex W.D.J.Koch in Rohling, Deutschlands Flora, ed. 3, 5: 68. 1839, isonym (Art. 6.3 Note 2 of the ICN) ≡ Polygala nicaeensis Risso, Fl. Nice: 54. 1844, isonym (Art. 6.3 Note 2 of the ICN) ≡ Polygala amblyptera var. nicaeensis (Risso ex Bray) Steud., Nomencl. Bot., ed. 2, 2: 370 1841, nom. illeg. (Art. 52.1 of the ICN).

##### Type.

France. “Alpes-Maritimes: “Esterel”, 15 May 1891, *G. Vidal* 2660 (first-step neotype, designated by Chodat 1893: 457; second-step neotype, designated here: G01100099! isoneotypes: BESA010305!, BR0000031351249!, CHE025420!, CLF17731!, G01100098!, G01100100!, P02998871!, P02999407!, P03209034!, P03209039!, P03209061!, MA-01-00074129).

While the name *P. nicaeensis* is universally attributed to Joseph Antoine Risso (1777–1845), the first description of this species is usually credited to Wilhelm Daniel Joseph Koch (1771–1849) as *P. nicaeensis* Risso ex W.D.J.Koch. However, some authors cite Koch (1836), others Koch (1839) and still others directly Risso (1844). Following further research into bibliographic sources that might link Risso and *Polygala* in publications prior to 1836, we were able to identify the place of valid publication of this species. It is found in a letter written by Franz Gabriel von Bray (1765–1832), published in 1818. In this letter, he reports on a journey he made to Nice in October of that year, during which he took two excursions, one with Augustin Pyramus de Candolle (1778–1841) and one with de Candolle and Risso. In the account of these excursions, he describes specimens of *Polygala* that Risso had named *Polygala nicaeensis* (Bray 1818). The correct scientific name for this species is, therefore, *P. nicaeensis* Risso ex Bray. This discovery re-defines the year and place of publication of this species, for which we have been unable to trace any original material. We searched for herbarium specimens collected by de Candolle or Risso in P, G-DC and NICE (Stafleu and Cowan 1976, 1983), finding a single specimen by Risso in G-DC!, dated 1837, which, therefore, does not belong to the original material. We also searched, without success, for specimens by von Bray in P, G-DC, NICE and REG, as he was, at that time, president of the Botanical Society of Regensburg (“Regensburgische Botanische Gesellschaft”). In the absence of original material and given the impossibility of designating a lectotype, the neotype already designated by Chodat (1893: 457) remains valid: the gathering no. 2660, Esterel 1891, *Vidal*. Since this gathering has been distributed in multiple duplicates across various European herbaria (BESA010305!, BR0000031351249!, CHE025420!, CLF17731!, G01100098!, G01100099!, G01100100!, P02998871!, P02999407!, P03209034!, P03209039!, P03209061!, MA-01-00074129), we proceeded with a second-step neotypification (Art. 9.17 of ICN), by restricting the neotype to the specimen in the Herbarium Delessert at G (G01100099!). The neotype matches the protologue and confirms the current application of the name as *P. nicaeensis* (Pignatti 2019). The question of whether *P. nicaeensis* s.str. and *P. vulgaris* subsp. pubescens Rhode ex Loisel. can be treated as heterotypic synonyms after their typification (see also below) requires further studies. This is particularly relevant because the latter name may apply to hairy, decumbent plants that were treated as “*P. nicaeensis*” by Polidori (2023), that may warrant some degree of taxonomic distinction. However, if these names are regarded as synonyms and treated at varietal rank, the priority name for this unit of systematic diversity is *Polygala vulgaris* subsp. pubescens Rhode ex Loisel., a name also cited by Steudel (1841) when he published the combination *Polygala amblyptera* var. nicaeensis (Risso ex Bray) Steud. For this reason, the latter epithet is illegitimate at varietal rank.

#### Polygala nicaeensis f. albiflora

Taxon classificationPlantaeFabalesPolygalaceae

Litard. in Briq. & Litard., Prodr. Fl. Corse 2(2): 61. 1936

1EC87552-0623-5ACC-8163-9A3643B1B59F

##### Type.

France. “maquis près de plage de Tamarone”, 18 May 1915, *R. de Litardière* s.n. (holotype, G00100146!).

Based on the morphological features of the holotype, also studied and mentioned by Jeanmonod (2010), *P. mediterranea* f. albiflora is a heterotypic synonym of *P. corsica* as circumscribed in this study.

#### Polygala nicaeensis f. coerulea

Taxon classificationPlantaeFabalesPolygalaceae

Briq. in Briq. & Litard., Prodr. Fl. Corse 2(2): 61. 1936

67AB8186-0AD9-53A3-988D-3AD80D15D07E

##### Type.

France. “de Solenzara à Porto-Vecchio, entre Ste Lucie et la Trinité, 80 m”, 4 May 1907, *J.I. Briquet, Saint-Yves et Cavillier* s.n. (lectotype, designated by Jeanmonod 2018: 68; G-BU [G00830051]!).

Based on the morphological features of the lectotype, we confirm *P. nicaeensis* f. albiflora as a heterotypic synonym of *P. corsica*, as circumscribed in this study. This confirms the interpretation of the type already made by Jeanmonod (2018), that considers this form as a heterotypic synonym of *P. pedemontana*, that is, in turn, a synonym of *P. corsica*, as circumscribed in this study.

#### Polygala nicaeensis var. commutata

Taxon classificationPlantaeFabalesPolygalaceae

Pomel, Bull. Soc. Sci. Phys. Algérie 11(7): 212. 1874

8EA50055-F0EE-5802-A774-30EC1DC9D2A4

Fig. 3

##### Type.

Algeria. “Chenoua”, 1874, *T. Clauson* s.n. (lectotype, designated here: MPU005108!).

**Figure 3.**
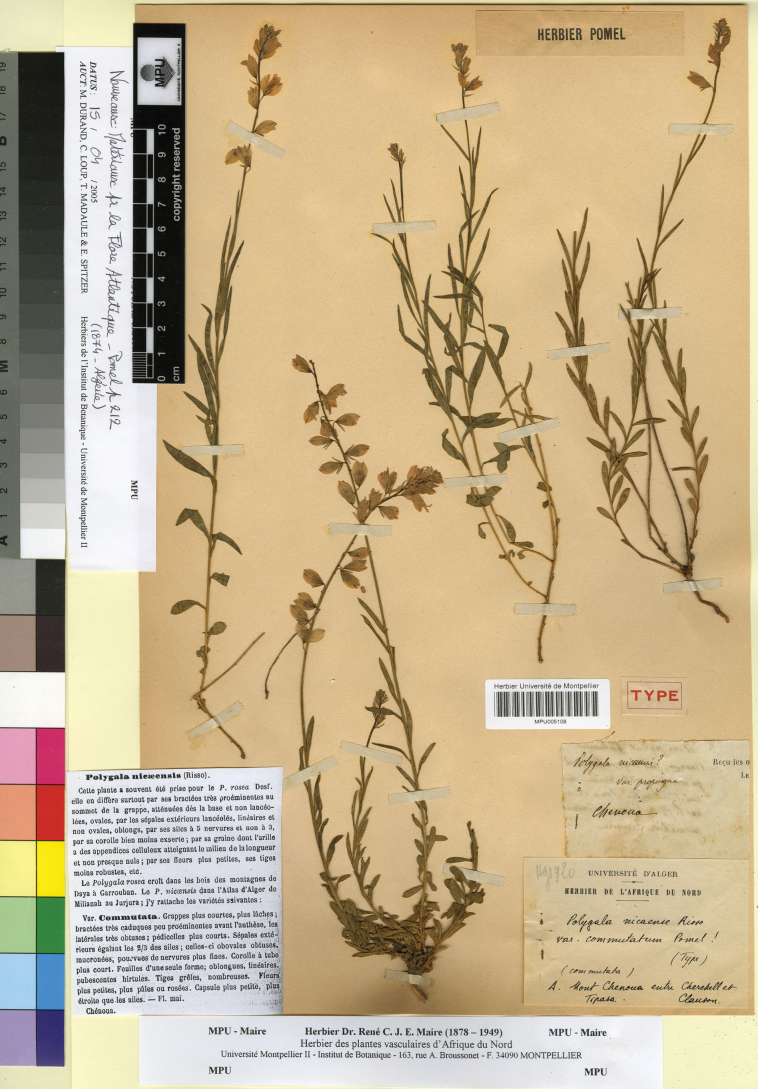
Lectotype of *Polygala nicaeensis* subsp. commutata Pomel in MPU. Reproduced with the permission of the Université de Montpellier – MPU Herbarium.

In the protologue (Pomel 1874), only “Chenoua” is indicated as collection source. We traced a specimen in MPU (MPU005108!) and another in P (P00375238) that are original material. We select the sample of MPU since it belongs to Pomel Herbarium. The lectotype matches the protologue. Based on the morphological features of the lectotype, *P. nicaeensis* subsp. commutata is a heterotypic synonym of *P. nicaeensis* s.str. as circumscribed in this study, i.e. a taxon combining the morphological features and distribution of the former *P. nicaeensis* subsp. nicaeensis and *P. nicaeensis* subsp. mediterranea Chodat.

#### Polygala nicaeensis var. corcyrensis

Taxon classificationPlantaeFabalesPolygalaceae

Halácsy, Consp. Fl. Graec. 1: 147. 1901

019B9280-17FE-55EA-BF74-9FCA2B26FF68

##### Type.

Greece. “prope urbem”, April 1890, *B. de Sagburg* s.n. (lectotype, designated here: WU0076374!).

In the protologue (von Halácsy 1901), the author mentions two specimens, a first one collected by B. de Sagburg in 1890 and a second one collected by C. Grimburg on 14 April 1897. According to Stafleu and Cowan (1979), the Herbarium von Halácsy is preserved in WU, where we found the two specimens (WU0076374! and WU0076375!, respectively), that are original material (see also Reich et al. (2021)). The second specimen, more complete, is selected as lectotype. The lectotype matches the protologue. Based on the morphological features of the lectotype, *P. nicaeensis* subsp. corcyrensis is a heterotypic synonym of *P. nicaeensis* s.str. as circumscribed in this study, i.e. a taxon combining the morphological features and distribution of the former *P. nicaeensis* subsp. nicaeensis and *P. nicaeensis* subsp. mediterranea Chodat.

#### Polygala nicaeensis f. albiflora

Taxon classificationPlantaeFabalesPolygalaceae

Chodat, Mém. Soc. Phys. Genève 31(2,2): 461. 1893

F455AD1B-2CE3-5CD4-B2C8-4C52E01C6BB0

##### Type.

ITALY. “In rupibus supra Sorrento (ai Conti)”, March 1841, *T. von Heldreich* s.n. (lectotype, designated here: P03244488!, individual on the left).

In the protologue (Chodat 1893), only Sorrento and some other localities are mentioned. By checking the herbaria consulted by Chodat (Chodat (1893), Preface page III, IV), we found a sheet with two different specimens mounted, each with its own label. The individual on the left is original material for the name, suitable for lectotypification. The lectotype matches the protologue. Based on the morphological features of the lectotype, *P. nicaeensis* f. elliptica is a heterotypic synonym of *P. nicaeensis* subsp. italiana (Arrigoni 2014), that, in turn, is a taxonomically doubtful subspecies (Bartolucci et al. (2024); see also the typification below).

#### Polygala nicaeensis f. heterophylla

Taxon classificationPlantaeFabalesPolygalaceae

Pamp., Boll. Mus. Rep. San Marino 4: 116. 1920

1F965BA7-AA80-5016-B966-ACE4A7B8DD12

##### Type.

San Marino. “Montalbo”, 4 May 1912, *R. Pampanini* 2115 (lectotype, designated here: FI-HCL-00695846!).

In the protologue (Pampanini 1920), no exact collection localities are mentioned. According to Stafleu and Cowan (1983), the Herbarium Pampanini is conserved in FI. There, six specimens were found (FI-HCL-00695845!, FI-HCL-00695846!, FI-HCL-00695847!, FI-HCL-00695848!, FI-HCL-00695851!, FI-HCL-00695852!) that are original material. In addition, two specimens were traced also in LY (LY0042331!, LY0042336!). The specimens in LY and the specimens FI-HCL-00695848, FI-HCL-00695851 are duplicates of the gathering no. 2489 of the “Flora Italica Exsiccata series 3”. The other four specimens are more specifically linked to the collections made by Pampanini for his flora of the Republic of San Marino. Amongst the latter specimens, we selected the most complete as lectotype. The lectotype matches the protologue. Based on the morphological features of the lectotype, *P. nicaeensis* f. heterophylla is a heterotypic synonym of *P. nicaeensis* subsp. italiana (Arrigoni 2014), that, in turn, is a taxonomically doubtful subspecies (Bartolucci et al. (2024); see also the typification below).

#### Polygala nicaeensis var. italiana

Taxon classificationPlantaeFabalesPolygalaceae

Chodat, Mém. Soc. Phys. Genève 31(2,2): 460. 1893

B7D6D11B-B2EB-5673-A4A0-261C70ABA0FC

≡ Polygala vulgaris subsp. italiana (Chodat) Fiori, Nuov. Fl. Italia 2: 123. 1925 ≡ Polygala nicaeensis subsp. italiana (Chodat) Arrigoni, Inform. Bot. Ital. 46: 246. 2014.

##### Type.

Italy. “sul Mt. pisano presso I bagni di S. Giuliano”, May, *P. Savi* s.n. (lectotype, designated here: P03244483!).

In the protologue (Chodat 1893), several localities from Tuscany (M. Summano, M. Pisano near San Giuliano Terme) and Val Perouse in Cottian Alps (a collection by Rostan) are mentioned. We traced several relevant specimens, all original material. We have examined the plate XXXIII fig. 41 (Chodat 1893) that depicts the shape of the seed and fruit of this variety, compared with others, that is original material as well. Neither specimens from Herbarium Rostan nor from M. Summano were found. On the contrary, we traced four specimens from San Giuliano Terme (P03244481!, BR0000031351331!, MPU755206!, P03244483!). The most complete of these specimens also includes a seed. Despite it lacks a precise collection year, it was included in P in 1875. The label was written by Pietro Savi (G. Astuti, pers. comm.). This specimen is selected as lectotype. Incidentally, we found in PI another specimen (PI 291502!), that could represent an isolectotype, but also bears the collection year (1849) on the label. The lectotype matches the protologue. Based on the morphological features of the lectotype, *P. nicaeensis* subsp. italiana is confirmed as a taxon endemic to Italy, currently accepted at subspecies rank (see also Arrigoni (2014)), marked by slightly longer capsule stipites, shorter bracteoles and caespitose habitus with respect to *P. nicaeensis* s.str. as circumscribed in this study. However, it should be highlighted that all these putative distinctive features are more or less overlapping between the two taxa, so that we totally agree with Bartolucci et al. (2024) in considering this subspecies as taxonomically doubtful and in need of further modern taxonomic studies.

#### Polygala nicaeensis subvar. laxa

Taxon classificationPlantaeFabalesPolygalaceae

Burnat, Fl. Alpes Marit. 1: 185. 1892

037A96FF-1A35-5CE2-B5E3-00B158DA0CE6

##### Type.

France. “Alpes-Maritimes: Environ de Nice”, 23 May, 10 June 1890, *G. Vidal* 2661 (lectotype, designated here: G01100101! isolectotypes: BR0000031351232!, BR0000031351683!, CHE025421!, CLF017730!, G00830213!, G01100101a!, G01100106!, LY00442297!, MPU628378!, P03013394!, P03209033! and P03209045!).

In the protologue (Burnat 1892), the author mentions three specimens and one gathering. According to Stafleu and Cowan (1976), we searched for these specimens in G. The gathering no. 2661 (“Flora selecta exsiccata”-published by C. Magnier) is duplicated on numerous specimens distributed in many French herbaria. All these specimens bear the annotation “vidit Burnat” and are original material. In particular, 17 specimens were traced, 13 of which are duplicates of the gathering no. 2661 (BR0000031351232!, BR0000031351683!, CHE025421!, CLF017730!, G00830213!, G01100101!, G01100101a!, G01100106!, LY00442297!, MPU628378!, P03013394!, P03209033! and P03209045!). We also traced four further specimens: the three in the Herbarium Burnat in G-BU (G00830222!, G00830219!, G00830214!), plus one duplicate in the Herbarium Burnat in MPU (MPU765489!). Amongst these materials, we selected the most complete specimen from gathering no. 2661 preserved in G (G01100101!) as lectotype. All the original materials match the protologue. Based on the morphological features of the lectotype, *P. nicaeensis* subsubsp. laxa is a heterotypic synonym of *P. nicaeensis* s.str. as circumscribed in this study, i.e. a taxon combining the morphological features and distribution of the former *P. nicaeensis* subsp. nicaeensis and *P. nicaeensis* subsp. mediterranea Chodat.

#### Polygala nicaeensis var. mauritanica

Taxon classificationPlantaeFabalesPolygalaceae

Chodat, Mém. Soc. Phys. Genève 31: 462. 1893

385BDDEE-EDF8-5861-AF29-2E565E6CEC7D

##### Type.

Algeria. “Costantine (Algérie): plateau du Télègraphe de Setif; abonde sur le pentes N.-O”, 21 May 1877, *V.C. Reboud* 1529 (lectotype, designated here: G01100103!, isolectotype: G01100102!).

In the protologue (Chodat 1893), gathering n. 1529 of the “Soc. Dauph” is mentioned. Two duplicate specimens in G were found (G01100102!, G01100103!) that are original material. The most complete specimen is selected as the lectotype. There, the fruits described by Chodat (1893) in the protologue as a diagnostic character are visible. Based on the morphological features of the lectotype, *P. nicaeensis* subsp. mauritanica is a heterotypic synonym of *P. nicaeensis* s.str. as circumscribed in this study, i.e. a taxon combining the morphological features and distribution of the former *P. nicaeensis* subsp. nicaeensis and *P. nicaeensis* subsp. mediterranea Chodat.

#### Polygala nicaeensis subsp. mediterranea

Taxon classificationPlantaeFabalesPolygalaceae

Chodat, Bull. Trav. Soc. Bot. Genève 5: 179. 1889

AA432F31-A3E9-5ED4-B757-6D5143F746B6

≡ Polygala nicaeensis subsp. insubrica Chodat, Bull. Trav. Soc. Bot. Genève 5: 179. 1889 (after typification) ≡ Polygala nicaeensis subsp. confusa Burnat, Fl. Alpes Marit. 1: 184. 1892, nom. illeg. (Art. 52.1 of the ICN) ≡ Polygala mediterranea (Chodat) Dalla Torre & Sarnth., Fl. Tirol 6(2): 763. 1909 ≡ Polygala vulgaris subsp. mediterranea (Chodat) O.Bolòs & Vigo, Butl. Inst. Catalana Hist. Nat., Secc. Bot. 38: 82. 1974.

##### Type.

France. “Forest Del’ Esterel prés Frejus”, 19 April 1861, *E. Bourgeau* s.n. (lectotype, designated here: G01100108!).

In the protologue (Chodat 1889), the author immediately lists three varieties under this subspecies: *P. nicaeensis* “subsp. graeca” nom. nud., *P. nicaeensis* var. corsica (Boreau) Chodat and *P. nicaeensis* subsp. insubrica Chodat, which he describes in detail. Within each of these varieties, he mentions a series of specimens, two in G for *P. nicaeensis* “subsp. graeca” (G00548641!, G00548640!), three from a Corsican collection by Sieber made in 1826 (BR0000031351263!, HAL112046!, COI00055844!) for *P. nicaeensis* var. corsica, to which the original material of *P. corsica* Boreau (ANG037772!, see above) should also be added. Finally, he mentions gatherings of *P. nicaeensis* subsp. insubrica from three localities (Lugano, Fort and Esterel), of which we have traced only two specimens collected in Esterel (G01100108!, G01100107!). From the study of all the traced original material, it can be concluded that the sample that is most suitable to be designated as the lectotype of *P. nicaeensis* subsp. mediterranea Chodat (≡ *P. nicaeensis* subsp. insubrica Chodat) comes from Esterel (G01100108!), since the only plants properly described by Chodat in the protologue (Chodat 1889) are those relating to this latter variety. The lectotype matches the protologue. Based on the morphological features of the lectotype, we can safely consider *P. nicaeensis* subsp. mediterranea as a heterotypic synonym of *P. nicaeensis* s.str. Moreover, after typification, these two taxa also share the same type locality (Esterel). This fully confirms the view expressed by Tison and de Foucault (2014), according to which *P. nicaeensis* subsp. nicaeensis and *P. nicaeensis* subsp. mediterranea would just represent the same diversity group, just differently described on young specimens of small size or on mature specimens of larger size.

#### Polygala nicaeensis var. obtusata

Taxon classificationPlantaeFabalesPolygalaceae

Pomel, Bull. Soc. Sci. Phys. Algérie 11(7): 213. 1874

5D612D17-5B00-5BCC-94A5-EB334219FE27

Fig. 4

##### Type.

Algeria. “A. Miliana”, s.d, *A.N. Pomel* s.n. (lectotype, designated here: MPU005109!).

**Figure 4.**
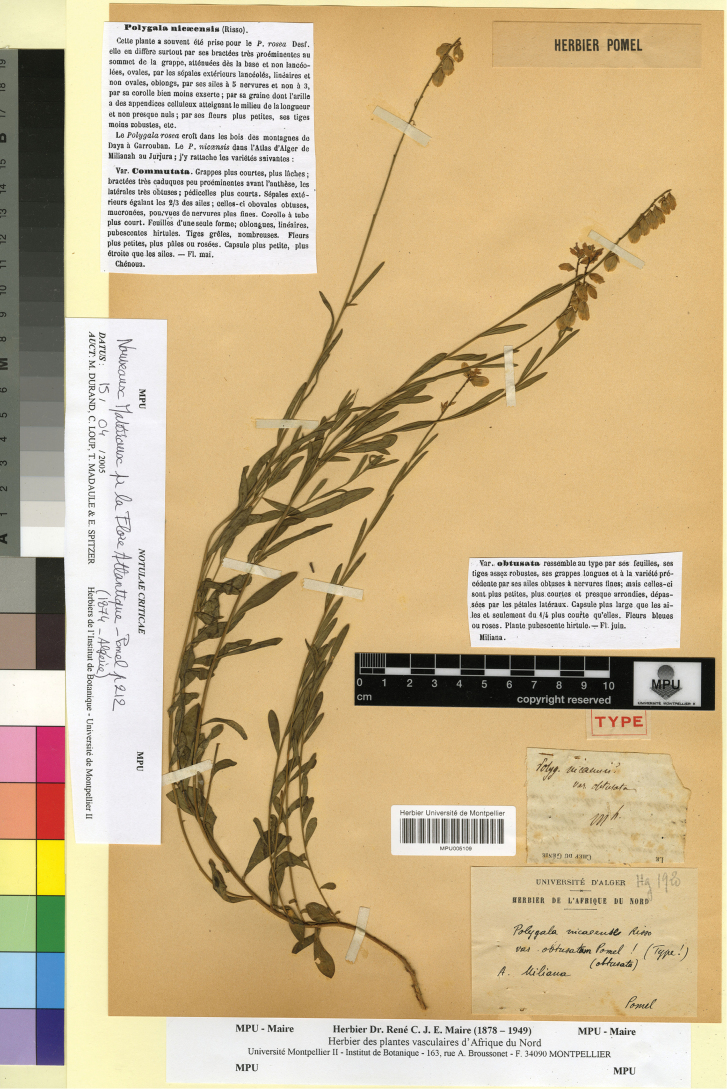
Lectotype of *Polygala nicaeensis* subsp. obtusata Pomel in MPU. Reproduced with the permission of the Université de Montpellier – MPU Herbarium.

In the protologue (Pomel 1874), only the collection locality is provided. We found only one specimen in MPU that can be considered as original material suitable for lectotypification. The lectotype matches the protologue. Based on the morphological features of the lectotype, *P. nicaeensis* subsp. obtusata is a heterotypic synonym of *P. nicaeensis* s.str. as circumscribed in this study, i.e. a taxon combining the morphological features and distribution of the former *P. nicaeensis* subsp. nicaeensis and *P. nicaeensis* subsp. mediterranea Chodat (see above).

#### Polygala nicaeensis subsp. peninsularis

Taxon classificationPlantaeFabalesPolygalaceae

Arrigoni, Inform. Bot. Ital. 46: 246. 2014

E509FBAA-532F-5C13-B894-70C154957F58

##### Type.

Italy. “Prov. Di Firenze, Mugello Giovigiana, suolo argill.-siliceo, alt. m. 700–900, 6 June 1922, *A. Fiori* s.n. (holotype: FI007309!)

Based on the morphological features of the holotype, *P. nicaeensis* subsp. peninsularis is confirmed as a subspecies endemic to Peninsular Italy (Arrigoni 2014), although considered as taxonomically doubtful by Bartolucci et al. (2024).

#### Polygala nicaeensis var. stenoptera

Taxon classificationPlantaeFabalesPolygalaceae

Chodat, Mém. Soc. Phys. Genève 31(2,2): 458. 1893

2C206C8E-52B2-5CDF-BE8E-B1DCAA8CB064

Fig. 5

##### Type.

France. “Nice”, June 1837, *A. Risso* s.n. (lectotype, designated here: G01094281!).

**Figure 5.**
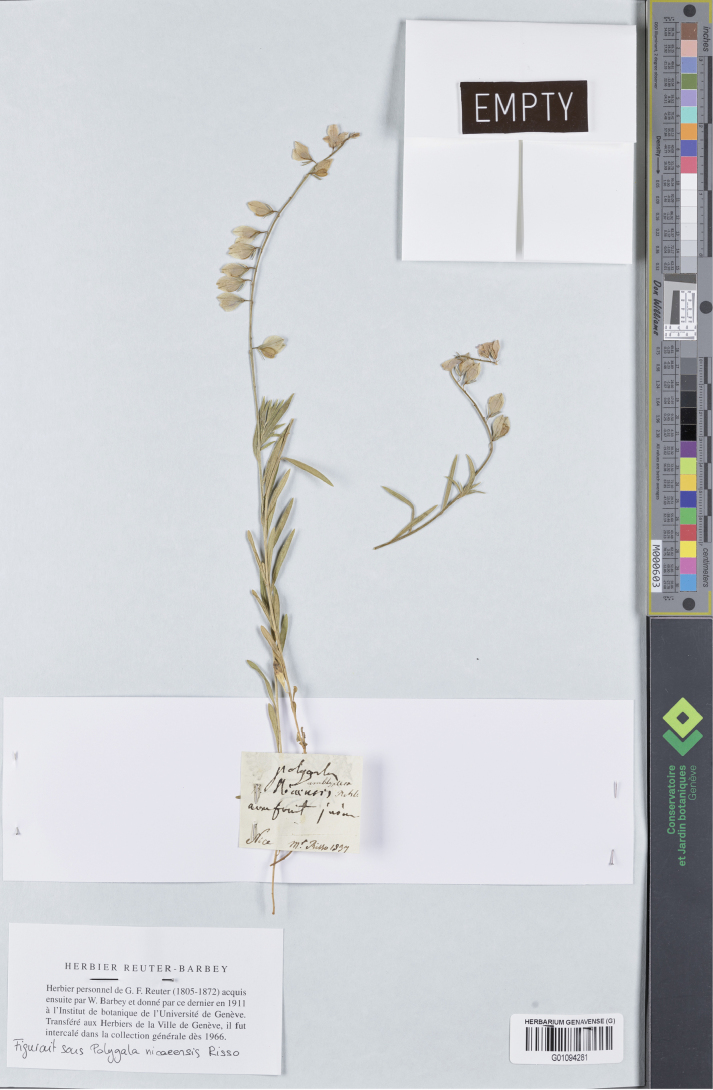
Lectotype of *Polygala nicaeensis* subsp. stenoptera Chodat in G. Reproduced with the permission of Genève Herbarium Conservatoire et Jardin botaniques de Genève.

In the protologue (Chodat 1893), two specimens collected by Risso and by Reuter, respectively, are mentioned. We traced only one sample in G that can be considered original material suitable for lectotypification. The lectotype matches the protologue. Based on the morphological features of the lectotype, coming from the same locality as the type of *Polygala nicaeensis* subsubsp. laxa Burnat (see above), also *P. nicaeensis* subsp. stenoptera is a heterotypic synonym of *P. nicaeensis* s.str. as circumscribed in this study, i.e. a taxon combining the morphological features and distribution of the former *P. nicaeensis* subsp. nicaeensis and *P. nicaeensis* subsp. mediterranea Chodat (see above).

#### Polygala nicaeensis var. tomentella

Taxon classificationPlantaeFabalesPolygalaceae

Boiss., Fl. Orient. 1: 475. 1867

F8D087ED-4F5E-5B1D-B260-9CB104EEBA59

≡ Polygala nicaeensis subsp. tomentella (Boiss.) Chodat, Bull. Trav. Soc. Bot. Genève 5: 179. 1889 ≡ Polygala nicaeensis subsp. graeca Chodat, Mém. Soc. Phys. Genève 31(2, 2): 463. 1893, nom. illeg. (Art. 52.1 of the ICN).

##### Type.

Greece. “Ad basin montis Parnis Atticae”, May 1842, *E. Boissier* s.n. (lectotype, designated here: [G-BOIS] G00150172!).

In the protologue (Boissier 1867), five herbarium specimens with location and collector are mentioned. We traced all these specimens in G-BOIS. The gathering by Orphanides has several duplicated in G-BOIS (G00548641!, G00150165!, G150177!, G00150179!), while other collections are represented each by a single specimen (G00150170!, G00150172!, G00150171!, G00150176!, G00150178!). Despite all these specimens being original material, only three show the tomentosity and occurrence of bristles mentioned in the protologue (“undique tomentello-hirtula”): G00150171, G00150172, G00150176. Amongst them, we selected here the most complete specimen. *Polygala nicaeensis* subsp. tomentella is currently considered as a subspecies endemic to Greece (McNeill 1968; Strid 1986). In particular, McNeill (1968) uses as a putative character to distinguish this subspecies its tomentosity, while Strid (1986) refers to straight vs. curved hairs, respectively, to distinguish *P. nicaeensis* subsp. tomentella from *P. nicaeensis* subsp. mediterranea, also occurring in Greece. Based on the morphological features of the lectotype, we confirm these views and preliminarily accept this taxon. However, considering that even Boissier (1867) mixed such kind of specimens with others showing a different pubescence, similar to that shown by *P. nicaeensis* s.str. as circumscribed in this study, further studies are necessary to understand whether such kind of peculiar indumentum has taxonomic value.

#### Polygala numidica

Taxon classificationPlantaeFabalesPolygalaceae

Pomel, Bull. Soc. Sci. Phys. Algérie 11(7): 211. 1874

B97943A2-59AC-5B40-98CD-557D3DB64F51

Fig. 6

##### Type.

ALGERIA. “La Calle”, s.d., *Hagenmüller* s.n. (lectotype, designated here: MPU005111!).

**Figure 6.**
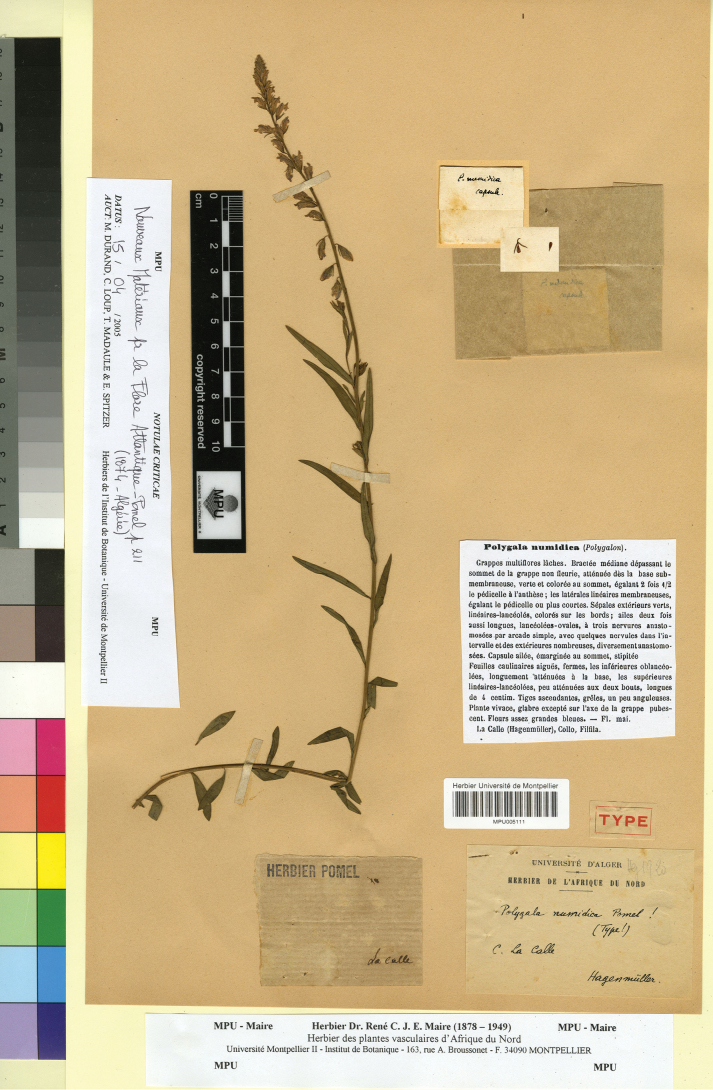
Lectotype of *Polygala numidica* Pomel in MPU. Reproduced with the permission of the Université de Montpellier – MPU Herbarium.

In the protologue (Pomel 1874), a single specimen (“La Calle (Hagenmüller), Collo, Filfila”) is mentioned. We traced at MPU this specimen, that is original material suitable for lectotypification. The lectotype matches the protologue. Based on the morphological features of the lectotype, *P. numidica* is a heterotypic synonym of *P. nicaeensis* s.str. as circumscribed in this study, i.e. a taxon combining the morphological features and distribution of the former *P. nicaeensis* subsp. nicaeensis and *P. nicaeensis* subsp. mediterranea Chodat (see above).

#### Polygala pedemontana

Taxon classificationPlantaeFabalesPolygalaceae

E.P.Perrier & B.Verl., Rev. Hort. (Paris) 12: 433. 1863

9DA72F14-9EEB-5A2A-AA1D-3E6B8AA405FA

≡ Polygala comosa var. pedemontana (E.P.Perrier & B.Verl.) Chodat in Burnat, Fl. Alpes Marit. 1: 188. 1892 ≡ Polygala comosa subsp. pedemontana (E.P.Perrier & B.Verl.) P.Fourn., Quatre Fl. France: 634. 1936 ≡ Polygala vulgaris subsp. pedemontana (E.P.Perrier & B.Verl.) Rouy in Rouy & Foucaud, Fl. France 3: 70. 1896 ≡ Polygala vulgaris var. pedemontana (E.P.Perrier & B.Verl.) Paol. in Fiori & al., Fl. Anal. Italia 2: 231. 1901 ≡ Polygala nicaeensis subsp. pedemontana (E.P.Perrier & B.Verl.) B.Bock, Bull. Soc. Bot. Centre-Ouest 43: 216. 2012.

##### Type.

France. “Mont-Cenis au dessous De la Poste (Italie)”, 2 August 1863, *E. Perrier de la Bathie* s.n. (lectotype, designated here: G01100104!).

In the protologue (Perrier and Verlot 1863), the authors mention an excursion on “Mont Cenis” without citing herbarium collections. According to Stafleu and Cowan (1983), the herbarium materials of these authors are conserved in G. There, we traced three specimens (G01100104!, G01100105!, G01100097!) that are original material. The specimen selected as lectotype is the most complete and corresponds well to the description in the protologue. Based on the morphological features of the lectotype, *P. pedemontana* is a heterotypic synonym of *P. corsica*, just showing slightly wider wings (see the typification above of the latter species, for further details). Despite this synonymy being already supported by Jeanmonod and Gamisans (2013) and by Tison and de Foucault (2014), these authors retained the name *P. pedemontana* as accepted. Despite this latter name being accepted also by other recent authors (Arrigoni 2014; Juillerat et al. 2017; Pignatti 2018; Polidori 2021; Bartolucci et al. 2024), the correct priority name for this species is actually *P. corsica*.

#### Polygala rosea var. occidentalis

Taxon classificationPlantaeFabalesPolygalaceae

Willk. in Willk. & Lange, Prodr. Fl. Hispan. 3(3): 558. 1878

984041CF-6829-55AD-AB83-E2E774217530

##### Type.

France. “collines a Draguignan (Var.)”, 10–21 April 1861, *E.S-C Cosson* s.n. (lectotype, designated here: COI00055847!).

In the protologue (Willkomm and Lange 1878), five specimens are mentioned: one originally labelled as *P. nicaeensis*, one as *P. corsica*, one as *P. gypsophiloides*, one as *P. vulgaris* and one as *P. saxatilis*. According to Stafleu and Cowan (1988), the Herbarium Willkomm is conserved at COI. There, we traced four herbarium specimens (COI00055844!, COI00055841!, COI00055846!, COI00055847!) that are original material. The specimen selected as lectotype is constituted by one individual in excellent conservation condition, in which many diagnostic characteristics can be evaluated such as the plant habitus, the presence of bracteoles in very young flowers, the size of the wings and of corolla tubes. The lectotype matches the protologue. Based on the morphological features of the lectotype, *P. rosea* subsp. occidentalis is a heterotypic synonym of *P. nicaeensis* s.str. as circumscribed in this study, i.e. a taxon combining the morphological features and distribution of the former *P. nicaeensis* subsp. nicaeensis and *P. nicaeensis* subsp. mediterranea Chodat (see above).

#### Polygala rosea var. orientalis

Taxon classificationPlantaeFabalesPolygalaceae

Willk. in Willk. & Lange, Prodr. Fl. Hispan. 3(3): 558. 1878

FCC2CD9F-1664-5653-91D4-7057A5681C5A

##### Type.

Greece. “In reg. Inferiori m. Parnethis pr. Zekeleiam”, 19 April 1876, *T. de Heldreich* 274 2245 (lectotype, designated here: COI00055854! isolectotypes: P02975640! P04200001! CLF335644! G-BOIS [G00150167]!).

In the protologue (Willkomm and Lange 1878), the fig. 51 by Reichenbach (1823) and one gathering by Heldreich (on which up to three numbers together are reported: Herbarium Graecum norm. n. 274, Herb, fl. Hellen. n. 71 and pl. exs. Graecia, n. 2245) are mentioned. We traced three herbarium specimens in COI (COI00055852!, COI00055854!, COI00055855!) and we found duplicates in P!, CLF! and G! that are original material. The most complete and best preserved specimen is selected as the lectotype. It is mounted with another specimen on the same sheet, where it occupies the upper part. The lectotype matches the protologue. Based on the morphological features of the lectotype, *P. rosea* subsp. occidentalis is a heterotypic synonym of *P. nicaeensis* subsp. tomentella (see above).

#### Polygala versicolor

Taxon classificationPlantaeFabalesPolygalaceae

Pomel, Bull. Soc. Sci. Phys. Algérie 12: 336–337. 1875

74FEBE48-935F-55EC-B279-3EA054F1BBFA

Fig. 7

≡ Polygala nicaeensis subsp. versicolor (Pomel) Batt. in Batt. & Trab., Fl. Algérie, 1: 106. 1888.

##### Type.

Algeria. “ras pharaon”, 18 June 1874, *A.N. Pomel* s.n. (lectotype, designated here: MPU005803!).

**Figure 7.**
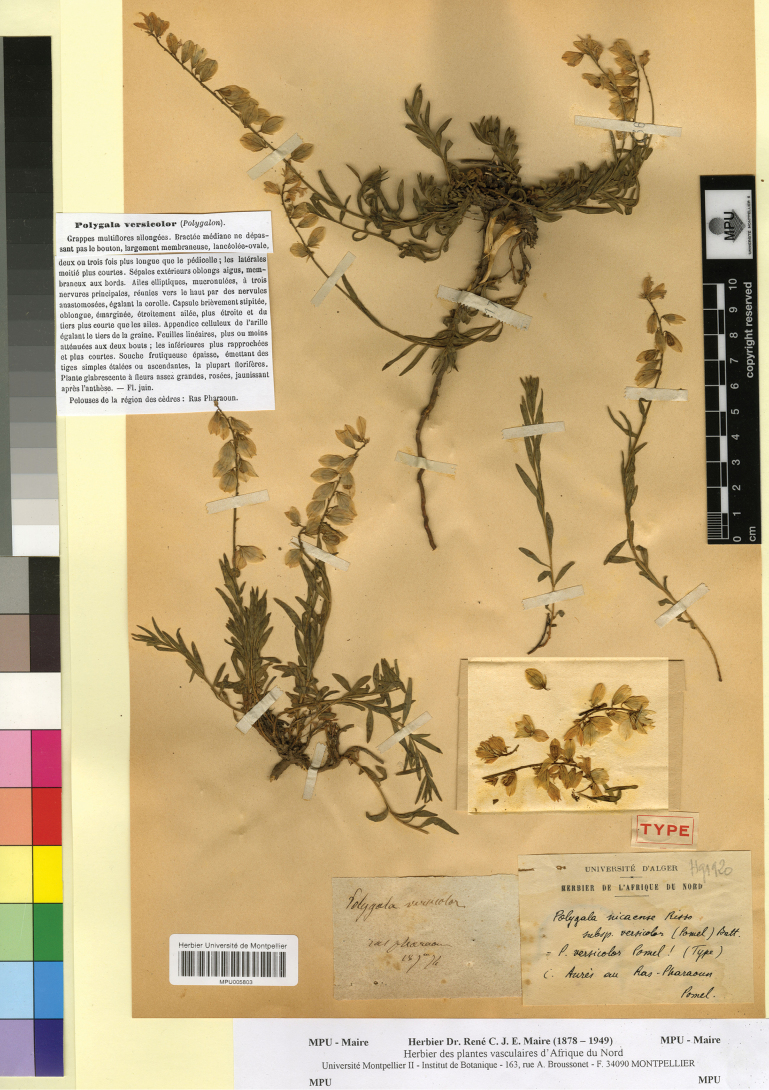
Lectotype of *Polygala versicolor* Pomel in MPU. Reproduced with the permission of the Université de Montpellier – MPU Herbarium.

In the protologue (Pomel 1875), only the locality “Pelouses de la région des cèdres: Ras Pharaoun” is mentioned. We found two specimens, one in MPU (MPU005803!) and one in P (P00375237!) that are original material. We selected the specimen of MPU as lectotype, because it also indicates the collection data on the label, missing in the P specimen. The lectotype matches the protologue. Based on the morphological features of the lectotype, *P. versicolor* is a heterotypic synonym of *P. nicaeensis* s.str. as circumscribed in this study, i.e. a taxon combining the morphological features and distribution of the former *P. nicaeensis* subsp. nicaeensis and *P. nicaeensis* subsp. mediterranea Chodat (see above).

#### Polygala vulgaris var. gerundensis

Taxon classificationPlantaeFabalesPolygalaceae

O.Bolòs & Vigo, Butl. Inst. Catalana Hist. Nat., Secc. Bot. 38(1): 82. 1974

1FA8C824-EB1A-55A3-8079-FCBF3E993AAD

≡ Polygala vulgaris subsp. gerundensis (O.Bolòs & Vigo) O.Bolòs, Vigo, Masalles & Ninot, Fl. Manual Països Catalans: 1215. 1990 ≡ Polygala nicaeensis subsp. gerundensis (O.Bolòs & Vigo) Á.M.Hern., Estud. Fl. Sant Llorenç del Munt: 148. 1993 ≡ Polygala nicaeensis subsp. gerundensis (O.Bolòs & Vigo) Mateo & M.B.Crespo, Fl. Abrev. Comun. Valenciana: 430. 1995, isonym (Art. 6.3 Note 2 of the ICN).

##### Type.

Spain. “Prov. De Gerona: Robledales de Maçanes, sobre granito 80 m alt.”, 4 June 1947, F.Q. s.n. (holotype: BC104488!).

Incidentally, Paiva (2007) wrongly considers *P. nicaeensis* subsp. caesalpini an illegitimate name and accepts this name for this subspecies. Based on the morphological features of the holotype, we can safely consider *P. nicaeensis* subsp. gerundensis as a heterotypic synonym of *P. nicaeensis* subsp. caesalpini. In addition, after the typification the latter name, these two taxa also share the same type locality (Gerona).

#### Polygala vulgaris var. pubescens

Taxon classificationPlantaeFabalesPolygalaceae

Rhode ex Loisel., J. Bot. 2: 359. 1809

620A063F-DDF8-509C-9218-C415A1C602F9

≡ Polygala buxifolia subsp. pubescens (Rhode ex Loisel.) Rchb., Iconogr. Bot. Pl. Crit.: 26. 1823, nom. illeg. (Art. 52.1 of the ICN) ≡ Polygala nicaeensis subsp. pubescens (Rhode) Burnat, Fl. Alpes Marit. 1: 184. 1892.

##### Type.

FRANCE. “Nice”, 1807, *J.G. Rhode* s.n. (lectotype, designated here: G-DC [G00210574!]).

In the protologue (Loiseleur-Deslongchamps 1809), Nice is mentioned as type locality. Despite this author not being covered by Stafleu and Cowan (1983), Burnat (1892: 184) mentions that he saw a specimen by Rhode in G-DC. We traced this specimen, which is original material suitable for lectotypification. The lectotype matches the protologue. It is worth noting that Rhode’s name is mentioned in both the works by Koch (1836, 1839), so far considered as putative places of valid publication for the name *P. nicaeensis* by main nomenclatural databases (Euro+Med 2025; POWO 2025; WFO 2025) and previous authors (Arrigoni 2014). Unfortunately, this latter species was first described by Bray (1818) and, in that work, there is no mention at all of Rhode’s name. For this reason, these two names cannot be homotypic. Whether *P. vulgaris* subsp. pubescens Rhode ex Loisel. and *P. nicaeensis* s.str. can be treated as heterotypic synonyms after their typification remains uncertain. The former name, whose type is characterised by the presence of relatively long hairs (ca. 0.2 mm), may apply to hairy, decumbent plants with hypogeal stems that were treated as “*P. nicaeensis*” by Polidori (2023). Such plants, endemic to the Maritime Alps, were regarded by that author as distinct from “*P. mediterranea*”, whose individuals exhibit a more erect habit without hypogeal stems and are glabrous or bear shorter hairs (ca. 0.1 mm). The latter feature is shared by both the types of *P. nicaeensis* s.str. and *P. nicaeensis* subsp. mediterranea. This issue need further investigation. As previously noted in the account devoted to the typification of *P. nicaeensis* s.str., if *P. vulgaris* subsp. pubescens and *P. nicaeensis* s.str. are treated as heterotypic synonyms, *P. vulgaris* subsp. pubescens has priority and should be adopted for *P. nicaeensis* when the taxon is treated at the varietal rank.

## Supplementary Material

XML Treatment for Polygala caesalpini

XML Treatment for Polygala corsica

XML Treatment for Polygala coursiereana

XML Treatment for Polygala gariodiana

XML Treatment for Polygala mediterranea var. jordanovii

XML Treatment for Polygala mediterranea var. parilica

XML Treatment for Polygala nemorivaga

XML Treatment for Polygala nicaeensis

XML Treatment for Polygala nicaeensis f. albiflora

XML Treatment for Polygala nicaeensis f. coerulea

XML Treatment for Polygala nicaeensis var. commutata

XML Treatment for Polygala nicaeensis var. corcyrensis

XML Treatment for Polygala nicaeensis f. albiflora

XML Treatment for Polygala nicaeensis f. heterophylla

XML Treatment for Polygala nicaeensis var. italiana

XML Treatment for Polygala nicaeensis subvar. laxa

XML Treatment for Polygala nicaeensis var. mauritanica

XML Treatment for Polygala nicaeensis subsp. mediterranea

XML Treatment for Polygala nicaeensis var. obtusata

XML Treatment for Polygala nicaeensis subsp. peninsularis

XML Treatment for Polygala nicaeensis var. stenoptera

XML Treatment for Polygala nicaeensis var. tomentella

XML Treatment for Polygala numidica

XML Treatment for Polygala pedemontana

XML Treatment for Polygala rosea var. occidentalis

XML Treatment for Polygala rosea var. orientalis

XML Treatment for Polygala versicolor

XML Treatment for Polygala vulgaris var. gerundensis

XML Treatment for Polygala vulgaris var. pubescens

## Additional information

### Use of AI

No use of AI was reported.

### Author ORCIDs

Lorenzo Peruzzi https://orcid.org/0000-0001-9008-273X

### Data availability

All of the data that support the findings of this study are available in the main text.
